# Effect of dietary honey on intestinal microflora and toxicity of mycotoxins in mice

**DOI:** 10.1186/1472-6882-6-6

**Published:** 2006-03-14

**Authors:** Aly M Ezz El-Arab, Shenouda M Girgis, Eman M Hegazy, Azzat B Abd El-Khalek

**Affiliations:** 1 Department of Food Science and Nutrition, National Research Center, 12644 – Dokki, Giza, Egypt; 2 Department of Cell Biology, National Research Center, 12644 – Dokki, Giza, Egypt; 3 Department of Food Toxicology, National Research Center, 12644 – Dokki, Giza, Egypt; 4 Department of Dairy Science, National Research Center, 12644 – Dokki, Giza, Egypt

## Abstract

**Background:**

Bee honey is a functional food which has a unique composition, antimicrobial properties and bifidogenic effect. In order to assess whether honey can inhibit the toxic effect of mycotoxins, the present study was undertaken.

**Methods:**

Production of biomass and toxins by *Aspergillus parasiticus and Aspergillus ochraceus *were followed in media without and with honey. Although aflatoxins and ochratoxin A. were administrated to male Swiss albino mice up to 1 μg and 10 ng/kg body weight/day respectively. The experimental animals were fed diets without our with 10% honey for two months. The changes in colonic probiotic bacteria, determintal colon enzyme glucuronidases, and genotoxicity were followed.

**Results:**

Addition of 32% in its media increased the biomass of *A parasiticus*, while the biomass of *A. ochraceus *decreased and Ochratoxin A. was not produced. When the honey was added at the ratio of 32 and 48% in the medium. No relationship was found between mycelium weight and production of mycotoxins. Oral administration of aflatoxins (mixture   of B_1_, B_2_, G_1_ and G_2_) and Ochratoxin A. induced structural and numerical   chromosomal aberrations in bone marrow and germ cells of male mice, whereas,   honey treatment reduced the genotoxicity of mycotoxins.   Also both toxins induced histopathological changes in liver and kidney. Feeding on diet supplemented with honey improved the histopathological changes in case of aflatoxin group, but not in the case of ochratoxin A. group (except of kidney in two cases). No significant differences were found in the activity of colon β-glucuronidase between group fed diet with or without honey. On the other hand, the colon bifido bacteria and lactobacilli counts were increased markedly in group receiving diet supplemented with honey.

**Conclusion:**

Substituting sugars with honey in processed food can   inhibit the harmful and genotoxic effects of mycotoxins, and improve the gut   microflora.

## Background

Foods are no longer appreciated by consumers only in terms of its taste and immediate nutritional needs, but also in terms of its ability to provide specific health benefits. Functional foods became an important food sector promoting health benefits via functional ingredients in these products. Functional foods targeted towards improving the balance and activity of the intestinal milieu currently provide the largest segment of functional food market [1,2].

For a long time, it has been observed that honey can be used to overcome liver, cardiovascular and gastrointestinal problems [3]. Honey is a natural product with very complex chemical composition. It is composed primarily of fructose and glucose but also contains 4 to 5% fructooligosaccharides which serve as prebiotic agents [4]. It contains more than 180 substances, including amino acids, vitamins, minerals and enzymes [5]. The antimicrobial properties of hydrogen peroxide and non-peroxide components for honey were tested in several studies [6].

The occurrence of mycotoxins (aflatoxins and ochratoxin) contamination is global. It's estimated that one quarter of the world's crops are contaminated to some extent with mycotoxins, especially prevalent in developing countries. Generally, mycotoxins are commonly found in foods [7,8]. Mycotoxins are of great concern because of their detrimental effects on the health of humans and animals. *Aspergillus flavus *and *A. parasiticus *produce aflatoxins. Ochratoxin A was discovered as a metabolite of *Aspergillus ochraceus*. Of the Aspergillus toxins, only ochratoxin (a natural carcinogen and nephrotoxin) is potentially as important as aflatoxins (a natural carcinogen). Both of them are associated with both toxicity and carcinogenicity in human health. A leading figure in the risk assessment field ranked mycotoxins as the most important chronic dietary risk factor, higher than synthetic contaminants, plant toxins, food additives, or pesticide residues [7,9].

Food contaminants entering the body through the oral route are directly exposed to the action of gut microflora. Gastrointestinal (GI) microflora is increasingly being recognised as one of the factors that influence human health. This microflora is a dynamic equilibrium that may be altered by diet [10]. Normal healthy intestinal microflora contains many strains of lactic acid bacteria (LAB) some of, which have been isolated, ascribed several beneficial effects, and termed probiotic strains [11]. Several studies showed that colonic probiotic bacteria can remove mycotoxins via physical binding as a mechanism for mutagen removal [12-14]. In addition to, the severity of mycotoxins poisoning can be amplified by factors such as vitamin deficiency, caloric deprivation, alcohol abuse and infectious disease status [7].

In addition, there is evidence that colonic probiotic bacteria may positively modulate certain colon microbial enzymes that may play a role in diet-related diseases. Alteration of these parameters are indicative of protective effects against carcinogen. Probiotic bacteria bind mutagens *in vitro*, and also they prevent excretion of mutagens in humans. However, more research is necessary to determine the physiological significance of endogenous colonic probiotic bacteria [15].

Complete elimination of any natural toxin from foods is an unattainable objective. Nutritional science has been expanding the knowledge of how foods influence consumers in relation to specific health parameters.

Therefore, it is necessary to perform more in-depth studies to elucidate the potential of protective activities by honey, through its antifungal activities and reducing dietary-related chronic diseases, such as cancer.

To achieve this goal, we studied *in vitro*, the effect of honey on the growth of fungi and formation of mycotoxins by Aspergillus in foods. In addition, we studied the effect of honey *in vivo *in presence of mycotoxin on endogenous colonic probiotic bacteria, colon microbial enzymes, cell proliferation and genotoxicity.

## Methods

A monofloral honey (Cotton) was purchased from the Bee Research Center, Ministry of Agriculture, Cairo, Egypt. Peanuts (*Archis hypogalal*) with damage and observable contamination with fungi were obtained from peanut main supplier's sources in Egypt. Bacterial culture, deMann, Rogosa, Sharpe (MRS) medium (Oxoid, Hampshire, United Kingdom) for Lactobacilli. Tryptone Phytone Yeast (TPY) medium (Oxoid) for Bifidobacteria. ochratoxin A standard (Cat. No. 01877) were purchased from Sigma-Aldrich chemical company (St. Louis, U.S.A). Aflatoxins and ochratoxin A producing fungi, *A. parasiticus *(FRR 2748) and *A. ochraceus *(NRRL 3174) were obtained from Standards Association of Australia (North Sydney, NSW, Australia). Natural aflatoxins (B_1_, B_2_, G_1 _and G_2_) were extracted from peanuts [16] and its concentrations were determined by thin layer (TLC) and high performance liquid chromatography (HPLC) techniques [17].

The concentration of extracted aflatoxins B_1_, B_2_, G_1 _and G_2 _from peanuts were 211.42, 135.53, 368.10 and 99.72 μg/kg, respectively. The obtained extract was dissolved in 2-liter soybean oil and stored in the refrigerator until used.

### Investigating the effect of honey on mycotoxin production

*A. parasiticus *(FRR 2748) and *A. ochraceus *(NRRL 3174) were cultivated on potato dextrose agar for 10 days at 25°C until they were well sporulated. The spores were harvested in 10 ml of sterile 1% Tween 80 solution (V/V) to give 10^5 ^spores/ml. Flasks containing 500 ml of Yeast- Extract-Sucrose medium (YES) supplemented with zero, 32 and 48% honey were inoculated with 2.0 ml of this suspension incubated at 28°C for 21 days and then assayed for aflatoxins [18,19].

### Investigating the effect of honey on mycotoxin ingestion in mice

Forty-two male Swiss albino mice, approximately 7 weeks old and weighing 20 ± 3 g, were obtained from the Animal House Colony at the National Research Center, Cairo, Egypt. Animals were individually housed in stainless steel cages under controlled temperature (24–26°C), humidity (40–70%) and lighting conditions (12 hr light/dark cycle) and allowed free access to distilled water and basic diet (AIN-93 M) for 10 days as an adaptive period. The AIN-93 M powder contained, protein (14%), starch (62.07%), sucrose (10%), fat (4%), fiber (5%), mineral mixture (3.5%), vitamin mixture (1%), L- cystine (0.18%), choline bitratrate (0.25%) and tetra-butylhydroquinone (0.00008%) [20].

The mice were then divided randomaly into six groups (7 animals each). The 1^st ^group received AIN-93 M diet and administered ochratoxin A (10 ng/kg bw/day, OT group). The 2^nd ^group were treated the same as group 1 except that it received honey/AIN-93 M diet (HOT group). Group 3 (AT) and H (HAT) were fed AIN-93 M and honey/AIN-93 M diet respectively and administered aflatoxins mixture (1 μg/kg bw/day). Group 5 (NTC) and 6 (HTC) served as controls and were fed AIN-93 M and honey AIN-93 M diet respectively. The experiment was carried out for 2 months, and animals were weighed weekly to calculate the daily dose. Animals were injected intraperitoneally with 0.5 ml of 0.05 % colchicine 2 hours before sacrifice to arrest the cells of bone marrow and spermatocyte in the metaphase stage following an overnight fast. The caecum contents were collected in clean Eppendorf tubes. Femur, liver, kidney, spleen, lung, testis and brain were removed.

### Activities of colon microbial enzymes

Bacterial β-glucuronidase activity was assayed in the caecum content digesta with P-nitrophenyl-β-D-glucuronide (Fluka, product No. 73677) [21]. Caecum dilution was performed at 4°C. The reaction mixture contained 0.3 ml substrate solution (5 mol P-nitrophenyl-β-D-glucuronide/l) and 0.2 ml of the diluted caecum (1 part of caecum and 2 parts phosphate buffer pH 6.4, w/v) and mixed well, incubated at 37°C/48 hrs. The concentration of released P-nitrophenol was measured from to the optical absorption at 400 nm after addition of 2.5 ml NaOH 0.1 N to stop reaction. Enzyme activity was expressed as mol product formed/g caecum content.

### Chromosomal aberrations examination

Chromosomes were prepared, stained with 5% Giemsa stain in phosphate buffer (pH 6.8), and 50 metaphase spreads were analyzed per animal for chromosomal aberrations in bone marrow cells [22]. Also chromosomal aberrations in germ cells were examined from testis of the same animals [23].

### Histopathlogical examination

Biopsies from liver, kidneys, spleens, lungs and brain were fixed in 10% buffered formalin solution for 12 hrs and processed into paraffin blocks. Five-mm thick tissue sections were cut on albuminzed glass slides and stained with haematoxylin and eosin stain (Hx. & E.). For proper evaluation of tissue fibrosis and better demonstration of blood vessels in tissue sections, massons trichrome stain was also used [24].

### Colonic probiotic bacteria investigations

The endogenous populations of colonic probiotic bacteria (Lactobacilli and Bifidobacteria) were counted. Caecum content samples were collected at the end of the experiment from each animal. The caecum content samples were immediately (within 30 min) placed in an anaerobe jar and kept at 4°C until analysed (a maximum of 6 hrs). Hundred fold serial dilutions were performed in pre reduced Ringer solution containing 0.5% of cystein. Petri dishes of various media were inoculated and incubated for 72 hrs at 37°C in anaerobic atmosphere using Gen Kits in Oxoid jars. Bacteria were detected on selective media as follows: deMan, Rogosa, Sharpe (MRS) medium (Oxoid, Hampshire, United Kingdom) for Lactobacilli and Tryptone Phytone Yeast (TPY) medium (Oxoid) for Bifidobacteria. After incubation, the colonies were counted. Bacterial counts are expressed as log_10 _colony forming units (log_10_CFU/g) of fresh caecum content sample, with detection limit 3.30 log_10_CFU/g.

### Statistics

All data were expressed as mean ± standard error (SEM) of the mean and analyzed by one way analysed of variance ANOVA using the SAS statistical programme (SAS version 6.11, SAS Institute, Cary, NC). When significant, Duncan's Multiple Range test was used to compare differences between means. The levels of significance were checked.

## Results

### Effect of honey on mycotoxin production

The *in vitro *investigations have shown that the biomass of *A. parasiticus *was enhanced in the medium containing 32% honey. However, biomas of *A. ochraceus *was decreased in medium containing 32 and 48% honey. Ochratoxin A was not produced at either honey concentrations. On the other side, aflatoxins B_1_, B_2_, G_1 _and G_2 _production was increased in medium containing 32% honey but decreased in medium of high honey concentration with no production of aflatoxin B_2 _and G_2 _(Table 1). Generally we did not find any positive relationship between mycelium weight and mycotoxins production.

**Table 1:** Effect of honey on mycelium mass and mycotoxins concentration.

Honey %	Mycelium weight (g)		Mycotoxins concentration (μg/l)				
	
	FRR 2748	NRRL 3174	Aflatoxins				Ochratoxin A
				
			B1	B2	G1	G2	
0	25.71	14.69	12.69	8.99	7.70	5.00	32.03
32.10	40.40	13.60	15.00	12.34	10.60	6.18	00.00
48.15	30.00	10.00	5.80	00.00	1.18	00.00	00.00

### Effect of honey on colonic glucuronidases activity

The effects of endogenous colonic probiotic bacteria on bacterial β-glucuronidases activity in the caecum content are shown in table 2. The level of glucuronidases was very low and no significant differences were observed in its in caecum content between all groups (P < 0.05).

**Table 2:** Effect of various mycotoxin and honey treatments on bacterial glucuronidases activity in the caecum content of mice.

β-glucuronidases μmol/g	Groups					
	
	OT	HOT	AT	HAT	NTC	HTC
	0.39 ± 0.11	0.40 ± 0.12	0.32 ± 0.10	0.37 ± 0.10	0.38 ± 0.14	0.42 ± 0.13

### Genotoxic effects of mycotoxins on bone marrow cells

**Figure 1:**
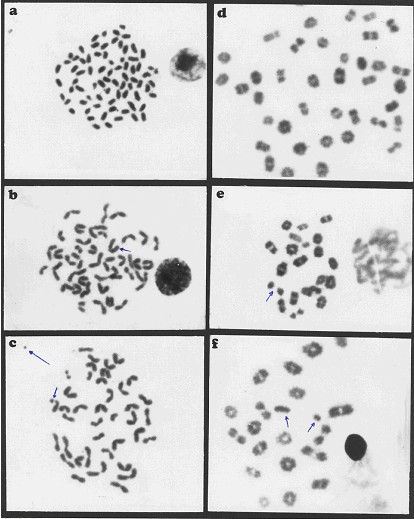
Metaphase spreads of male mice treated with ochratoxin A and aflatoxins showing: a) centromeric attenuation. b) deletion. C) chromatid break (small arrow), fragment (large arrow) of bone marrow cells and d) polyploidy. e) autosomal univalent. F) x-y univalent, of spermatocyte cells.

The present study revealed that the oral administration of aflatoxins (B_1_, B_2_, G_1 _& G_2_) and ochratoxin A to male mice induced structural and numerical chromosomal aberrations (Table 3 & Figure 1).

**Table 3:** Mean values of different types of chromosomal aberrations in bone marrow cells of male mice treated with ochratoxin A and aflatoxins (B_1_, B_2_, G_1 _and G_2_).

Treated groups	Numerical aberrations	Structural aberrations					
	
	Peridiploidy	Chromtatid gaps	Chromatid breaks	Centromeric attenuations	Deletions	Fragments	Total
OT	2.50 ± 0.45^a^	0.67 ± 0.17^a^	0.67 ± 0.21^a^	0.83 ± 0.17^b^	2.50 ± 0.00^a^	0.83 ± 0.21^a^	5.50 ± 0.31^b^
HOT	1.83 ± 0.33^a^	0.50 ± 0.00^a^	0.50 ± 0.00^a^	0.50 ± 0.00^b^	0.83 ± 0.21^b^	0.67 ± 0.17^a^	3.00 ± 0.34^c^
AT	2.83 ± 0.67^a^	0.67 ± .017^a^	0.67 ± 0.17^a^	4.33 ± 1.31^a^	1.17 ± 0.21^b^	0.83 ± 0.21^a^	7.67 ± 0.48^a^
HAT	2.50 ± 0.36^a^	0.50 ± 0.00^a^	0.50 ± 0.00^a^	0.67 ± 0.17^b^	1.00 ± 0.22^b^	0.50 ± 0.00^a^	3.17 ± 0.21^c^
NTC	2.00 ± 0.22^a^	0.50 ± 0.00^a^	0.50 ± .0.00^a^	0.50 ± 0.00^b^	0.83 ± 0.21^b^	0.50 ± 0.00^a^	2.83 ± 0.21^c^
HTC	2.00 ± 0.34^a^	0.50 ± 0.00^a^	0.50 ± 0.00^a^	0.50 ± 0.00^b^	0.67 ± 0.17^b^	0.50 ± 0.00^a^	2.67 ± 0.17^c^

- Means with different superscript letters (a, b, c) are significantly different (P ≤ 0.05).

-All data are expressed as means ± SEM.

Structural chromosomal aberrations were recorded as chromatid gaps, chromatid breaks (Figure 1c), centromeric attenuations (Figure 1a), deletions (Figure 1b) and fragments (Figure 1c). Table 3, represents the mean values of different structural chromosomal aberrations induced by mycotoxins in bone marrow cells of male mice. The results show low frequency in chromatid gaps, breaks and fragments, whereas the frequency of centromeric attenuation were significantly high between AT group and HAT group from one side and between AT group and the control groups (NTC and HTC) from the other side.

A significant difference (P < 0.05) was also found in deletions (Figure 1b) between OT group and HOT group, also between OT group and the control groups (NTC and HTC), which can be attributed to the protective effect of honey.

Significant increase (P < 0.05) in the frequency of total structural chromosomal aberrations was found between OT group and HOT group from one side and between group OT and the control groups (NTC and HTC) from another side. Also, a significant difference was found between AT group comparing to HAT group and the control groups (NTC and HTC), respectively.

### Genotoxic effects of mycotoxins on germ cells (spermatocytes)

The results of our study revealed that oral treatment with ochratoxin A and aflatoxins (B_1_, B_2_, G_1 _& G_2_) induced numerical and structural chromosomal aberrations in germ cells of male mice (Table 4). Numerical aberrations were recorded as peridiploidy (n ± 1 or n ± 2) and polyploidy (Figure 1d). Peridiploidy observed in spermatocytes of male mouse was clearly different (p ≤ 0.5) only in the aflatoxins treated group (AT) compared to the control groups (NTC and HTC). However, polyploidy induced by ochratoxin A treated group (OT) differed significantly (P < 0.05) when compared to the control groups. Meanwhile, the total numerical aberrations were significantly different (P < 0.05) in both ochratoxin A (OT group) and aflatoxins treated group (AT) in comparison to the control groups (NTC and HTC). The protective effect of honey was apparent only in HAT group.

**Table 4:** Mean values of different types of chromosomal aberrations in spermatocyte cells of male mice treated with ochratoxin A and aflatoxins (B_1_, B_2_, G_1 _and G_2_).

Treated groups	Numerical aberrations			Structural aberrations		
	
	Peridiploidy	Polyploidy	Total	X-Y univalents	Autosomal univalents	Total
OT	1.75 ± 0.48^ab^	3.75 ± 0.48^a^	5.25 ± 0.48^a^	2.75 ± 0.48^a^	1.00 ± 0.41^a^	3.75 ± 0.86^a^
HOT	1.50 ± 0.29^abc^	2.75 ± 0.25^a^	4.50 ± 0.65^ab^	1.00 ± 0.41^b^	0.75 ± 0.25^a^	1.75 ± 0.63^a^
AT	2.25 ± 0.25^a^	1.25 ± 0.25^b^	3.50 ± 0.29^b^	2.00 ± 0.92^ab^	0.75 ± 0.75^a^	2.75 ± 0.95^a^
HAT	1.50 ± 0.29^abc^	0.50 ± 0.50^b^	2.00 ± 0.0.41	1.50 ± 0.29^ab^	0.50 ± 0.29^a^	2.00 ± 0.58^a^
NTC	0.75 ± 0.48^bc^	1.00 ± 0.41^b^	1.50 ± 0.29^c^	1.50 ± 0.29^ab^	1.00 ± 0.41^a^	2.50 ± 0.29^a^
HTC	0.50 ± 0.29^c^	0.25 ± 0.25^b^	1.00 ± 0.41^c^	1.00 ± 0.14^b^	1.00 ± 0.00^a^	2. 00 ± 0.00^a^

Within a column, values with no common superscript are significantly different (P ≤ 0.05).

-All data are expressed as means ± SEM.

Treatment with ochratoxin A highly induced structural chromosomal aberrations especially x-y univalents (Figure 1f), where significant difference (P < 0.05) was found between OT and HOT groups, also between OT and the control groups (HTC).

### Effect of honey on cell proliferation

**Figure 2:**
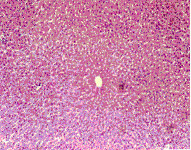
Liver of NTC group. Normal liver tissue (H x. & E. ×100).

Microscopic examination of the control liver tissue showed hexagonal hepatic lobules consisting of central vein from which hepatic cords, sinusoids lined by radiating kupffer cells as well as bile canaliculi were apparent. The portal tract was located between three hepatic lobules and consisted of portal vein, hepatic artery and bile duct (Figure 2).

**Figure 3:**
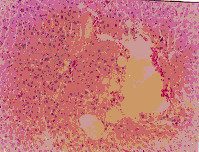
Liver of OT group. Dilated congested central veins marked sinusordal dilatation & congestion (Hx. & E. ×200).

**Figure 4:**
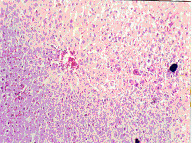
Liver of OT group. Focal hepatic necrosis predominantly peri vascular (Hx. & E. ×200).

**Figure 5:**
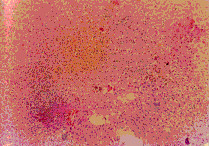
Liver of AT group. Variable-sized necrotic areas of the hepatic parenchyma infiltrated by neutrophils.(Hx. & E. ×100).

**Figure 6:**
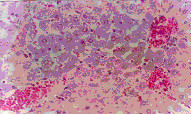
Liver of AT group. Congested dilated centeral veins and marked sinusoidal dilatation (Hx. & E. ×400).

**Figure 7:**
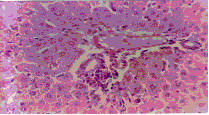
Liver of AT group. Portal tracts thickened by marked dilatation & congestion of vascular channels and mild mano-nuclear inflammatory infiltrate (Hx. & E. ×400).

Administration of ochratoxin A affected the liver tissue as it induced dilated congested central veins, with marked sinusoidal dilation, congestion and hyperplastic kupffer cells (Figure 4). Also focal hepatic necrosis, predominantly perivascular was evident (Figure 3). Administration of honey with ochratoxin A revealed no beneficial effect as the liver in AOT group was affected the same as in group OT. On the other hand, administration of aflatoxins caused cloudy swelling in hepatocytes, extensive variable side necrotic areas affecting the hepatic parenchyma and infiltrated by neutrophils and macrophages (Figure 5). Also, congested dilated central veins and marked sinusoidal dilatation with hupffer cell hyperplasia were also evident (Figure 6). The portal tracts are thickened by mononuclear inflammatory cells formed mainly of lymphocytes and telangiectatic, congested vascular channels (Figure 7).

**Figure 8:**
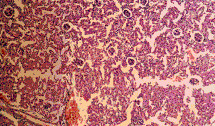
Kidney of OT group. Increased number & size of glomeruli (Hx. & E. ×100).

**Figure 9:**
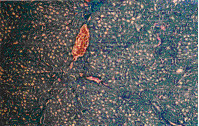
Kidney of OT group. Interstitial fibrosis and congestion (Massons trichrome ×100).

**Figure 10:**
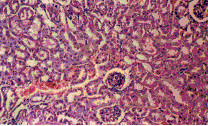
Kidney of HOT group. Return to normal except for vascular congestion (Hx. & E. ×200).

**Figure 11:**
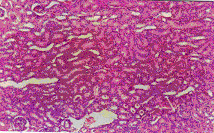
Kidney of AT group. Cloudy swelling of epithelium of tubules (Hx. & E. ×100).

Histopathlogical examination of the control kidney tissues showed glomeruli, proximal and distal convoluted renal tubules, the medulla contained the collecting tubules and parts of the ascending and dexcending loop of henle. Ochratoxin A administration induced cloudy swelling of proximal convoluted tubules, fibrin thrombin within glomerular capillary loops, increased number and size of glomeruli (Figure 8) and interstitial fibrosis and congestion (Figure 9). On the other hand, co-administration of honey with ochratoxin A ameliorated the effect of ochratoxin A on the kidney where vascular congestion returned to normal. However, they were still slightly enlarged (Figure 10). Administration of aflatoxins induced cloudy swelling of the epithelial lining of renal tubules with mild interstitial fibrosis and congestion (Figure 11).

**Figure 12:**
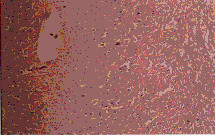
Brain. Focal areas of degeneration in the OT group (Hx. & E. ×100).

Regarding the effect of ochratoxin administration on brain, histopathlogical examination pointed to the presence of focal areas of degeneration (Figure 12), while Co-administration of honey with ochratoxin A ameliorated this effect. In addition, administration of aflatoxin did not induce any changes in brain tissue.

**Figure 13:**
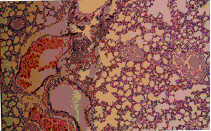
Lung of OT group. Edema, congestion, lymphoid aggregates of interstitial tissue (Hx. & E. ×100).

**Figure 14:**
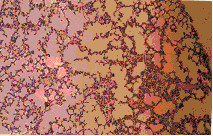
Lung of HOT group. Relief of congestion but edeme & lymphocytic aggregates are still present (Hx. & E. ×100).

**Figure 15:**
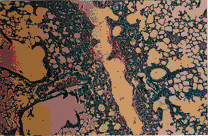
Lung of AT group. Showing interstitial fibrosis, inflammatory infillate with focal lymphocytic aggregates (Massons trichrome ×100).

Histopathlogical examination of the control lung showed thin alveolar walls, consisted of epithelial cells in both sides of centrally, located capillaries with no intervening connective tissue stroma. Administration of ochratoxin A affected the lung tissues where, the lung showed edema, congestion, lymphoid aggregates in interstitial tissue (Figure 13). The results pointed out that the lung showed relief of congestion (co-administration of honey with ochratoxin A) (Figure 14). Whereas, the lungs showed interstitial fibrosis and inflammatory infiltrate after administration of aflatoxin (Figure 15).

**Figure 16:**
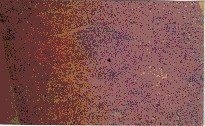
Spleen of NTC group. Induced similar histopathlogical, white & red pulps. (Hx. & E. ×100).

**Figure 17:**
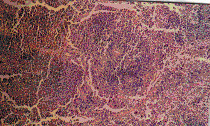
Spleen of OT group. Expansion of the white pulp with obliteration of normal architecture (Hx. & E. ×100).

The control spleen tissue showed connective tissue framework (which includes capsule, trabeculae and reticular connective tissue) and a parenchyma of lymphoid tissue in the form of white (lymphoid nodules) and red pulp (Figure 16). Figure 17 illustrated the effect of ochratoxin A on the spleen where it showed expansion of the white pulp with destruction of normal architecture, adventitial thickening of arterioles, adventitial thickening of arterioles focal fibrosis of the red pulp and compression of the sinusoids.

On the other hand administration of aflatoxins did not indicate any change in the spleen tissue. Co-administration of honey with aflatoxins causes improvement of all histopathologic changes that occurred in all examined organs except for kidney tissue.

### Effect of honey on colonic probiotic bacteria

Addition of honey to AIN-93 M powder diet (HTC group) increased the counts of bifidobacteria by a mean of 2.3 ± 0.23 log_10_CFU/g (p < 0.004) and lactobacilli by a mean of 1.07 ± 0.35 log_10_CFU/g (p < 0.016) in comparison to NTC group (Table 5).

**Table 5:** Effect of honey and mycotoxins on LAB growth (log_10_CFU/g).

Strains	Mice Groups					
	
	OT	HOT	AT	HAT	NTC	HTC
Lactobacilli						
	3.26 ± 0.19	3.83 ± 0.20	Nil	Nil	5.00 ± 0.15	6.06 ± 0.22
Bifidobacteria						
	3.73 ± 0.19	4.77 ± 0.41	3.20 ± 0.32	3.77 ± 0.22	6.26 ± 0.29	8.57 ± 0.24

Administration of ochratoxin A in to the group receiving honey (HOT group) increased the counts of lactobacilli by a mean of 0.75 ± 0.15 log_10_CFU/g (p < 0.108) in comparison to OT group. Lactobacilli were not detected in the case of aflatoxins administration (HAT & AT groups) (Table 5).

Administration of ochratoxin A and aflatoxins mixture in presence of honey (HOT & HAT groups) increased the mean counts of bifidobacteria by 1.03 ± 0.32 log_10_CFU/g (p < 0.083) and 0.57 ± 0.50 log_10_CFU/g (p < 0.219) in comparison to OT and AT groups, respectively.

## Discussion

### Effects of honey on mycotoxin production

Our results indicate that, the effect of honey on the biomass and mycotoxins formation (*in vitro*) was depend on the kind of fungi (*A. parasiticus *and *A. ochraceus*) and the concentration of honey. The antimicrobial properties of hydrogen peroxide and non-peroxide components for honey were reported in several studies [25]. Thus for these 2 types of fungi, honey neutralized more pathogens in dose-dependent fashion than did a control sugar solution (80%), consisting of fructose and glucose, similar to that in pure honey [25]. Undiluted honey inhibited completely the growth of common fungi of superficial infection and wound contamination (*Aspergillus fumicgatus, A flavus, Penicellum citrinum, Trichophyton rubrum *and *Candida albicans*), partial inhibition with honey concentration of 50% and no inhibition was recorded by honey concentration of 20% [26].

### Effect of honey on colonic probiotic bacteria

Lactobacilli and bifidobacteria have complex nutritional requirements such as carbohydrates, aminoacids, peptides, fatty esters, salts, nucleic acid derivatives, and vitamins, which vary markedly from species to species. Honey contains more than 180 substances, including amino acids, vitamins, minerals and enzymes [5]. The principal carbohydrate constituents of honey are fructose (32.56 %) and glucose (28.54 %). In addition to oligosaccharides (1.58 to 3.77% maltose, 0.78 ± 2.03% turanose, 1.11 ± 2.81% nigerose, 0.05 ± 0.15% meli-biose, 0.03 ± 0.08% panose, 0.24 ± 1.03% maltotriose, 0.21 ± 0.37% melezitose and 0.10 ± 0.25%. Honey also contains 4 to 5% fructooligosaccharides, which serve as probiotic agents [4].

The normal human small intestine mucosa may incompletely absorb fructose. Several studies have shown that prevalence of fructose malabsorption in normal adults may be as high as 30–50%, these subjects may malabsorb an appreciable amount of a 25 g-fructose dose [27]. The most normal subjects malabsorb about 10% of carbohydrates in honey [28]. Honey oligosaccharides with low degree of polymerization have been considered as favorable bifidobacterial substrate [10]. Finally, the non-digestible and/or the non-absorbable saccharides move to the colon to induce selective fermentation, and selective stimulation of the growth and activity of colonic probiotic bacteria as found previously [29].

The present study confirms the simulative effect of honey on colonic probiotic bacteria. The growth of intestinal Bifidobacteria spp. was enhanced with presence of more than lactobacilli spp, which may be due to the honey oligosaccharides with low degree of polymerization (4 to 5%).

The decrease in the count of colonic probiotic bacteria with the administration of mycotoxin and in absence or presence of honey may be due to the changes in the bacterial surface during growth phase, as a result of mycotoxins binding [13].

### Effect of honey on colon bacterial enzyme activities

The level of glucuronidases is an important surrogate for the effects of diet on the composition and activity of the intestinal microflora. It is involved in formation as well as inactivation of carcinogens in the gut lumen and may be altered in a positive way by the presence of colonic probiotic bacteria [30].

### The antigenotoxic effects of honey

Mycotoxins were found to be genotoxic to bone marrow and spermatocyte cells of mice, which coincide with previous reports [31,32], that the ochratoxin A and aflatoxins induced chromosomal aberrations in many mammalian cells (mouse, rat, Chinese hamster and even human being).

Using ochratoxin A and aflatoxins mixture as toxic and carcinogens, we have characterized the potential antigenotoxicity of honey. Honey was antigenotoxic (*in vivo*), and that effect was dependent on colonic probiotic bacteria and other factors. Colonic probiotic bacteria are proposed to have several beneficial effects, including the inactivation of carcinogen [15]. In addition to finding that colonic probiotic bacteria have antigenotoxic properties, it also immediate interest to study the mechanisms responsible for its protection. It has been suggested that a protein structure of bacterial lipopolysaccharides membrane could be an effective component [33]. Also, there have been several reports that lactic acid bacteria (LAB) can bind mutagens and thus may decrease mutagenicity [34].

Nutritional factors found in honey could be used to prevent and eliminate aflatoxins induced mutation [35]. This was in accordance with our findings, which revealed, could be prevent or reduce the chromosomal aberrations induced by aflatoxins. In addition, AFB1- induced DNA damage and chromosomal aberrations in rodent and human cells can be modulated by a variety of factors including nutrients and chemopreventive agents [36]. Those are in agreement with our results, where the administration of honey could improve the genetic materials and minimize the chromosomal aberrations induced by mycotoxins.

### Effect of honey on cell proliferation

The present results concerning the histopathological study revealed that ochratoxin A induced haemorrhage and necrosis of the red pulp of the spleen. This finding was in accordance with the work done by [30]. Furthermore, necrosis in the white pulp, involved cortical and medullry regions of lymphoid follicles. In addition, ochratoxin A is connected with an impairment in the structure and function of brain cell membrane [31]. It may increase, markedly the activities of the cytosolic and lysosomal enzymes.

In the present study, the effect of aflatoxins on the liver agrees with the work done before [32] whhich explained this affect to an increase in cytoplasmic easihophilia and loss of cytoplasmic granularity of hepatocytes. Also, perioprtal fibrosis with degeneration and ulceration of bile duet epithelial cell lining and proliferation of bile ducts were recorded [33]. Multifocal, randomly distributed areas of hepatic necrosis infiltrated with neutrophils and macrophages were evident. The lesions were central being more pronounced around portal and similar result [34]. The results of the present work are in accordance with previous reported [35], that indicating rats treated by intraperitoneal injection of Aflatoxin B_1 _showed pronounced inflammatory reaction in their lungs, congestion, destruction of alveolar walls with amphysematous changes and thickening of the walls of the blood vessels, occurring in patchy areas of the lung tissue.

From the histopathologic study it can be concluded that the addition of honey to ochratoxin A improved slightly the microscopic changes of kidney tissue only. However, addition of honey aflatoxins improved the histopathologic changes caused by the toxin, in different organs except for kidney tissue.

Our results indicated that, the detrimental effects of mycotoxins can be decreased in presence of honey, which may be via, the detoxification of mycotoxins by probiotic bacteria.In the same time, the accessibility of bound AFB1 to an antibody suggests that surface components of these bacteria were involved in toxin binding [13]. Variation in temperature (4 to 37°C) and pH (2 to 10) had no significant effect on the amount of AFB1 released. Binding of AFB1 appears to be predominantly extracellular for viable and heat-treated bacteria. However, acid treatment may permit intracellular binding. In all cases, binding is reversible, but the stability of the complexes formed depends on strain, treatment, and environmental conditions. Generally, detachment of AFB1, from the bacterial surface requires time. During that time, the AFB1 records down along the GI tract towards expulsion. This sequestration of AFB1 reduces the opportunity for it to be reabsorbed or exert a pathogenic effect on the enterocytes or gut-associated lymphoid tissue (GALT). This may be the net effect of detoxifying mycotoxins from the system. Also, our results are compatible with [36], as ochratoxin A exhibits cloudy swelling of proximal convoluted tubules in addition to tubular eosinohilic intraluminal proteinaceous casts, fibrin thrombi within glomerular capillary loops and marked interstitial fibrosis and congestion.

Aflatoxins are genotoxic carcinogens. For this type of carcinogens, it is generally felt that there is no threshold dose below which no tumor formation would occur. In other words, only a zero level of exposure will result in no risk. This agrees with the recent evaluation of [37], with respect to the genotoxicity of the aflatoxin [38]. It is necessary to perform in depth studies to elucidate the effect of growth phase of all probiotic bacteria strains on AFB_1 _and G_1 _binding and other mycotoxins *in vitro *and *in vivo*.

## Conclusion

The present results suggest that honey has protective effects dependant on its antimicrobial properties. Also honey enhances the endogenous colonic probiotic bacteria (bifidogenic effects) that has several beneficial effects (i. e. detoxification and antigenotoxicity).

We recommend that substituting sugars with honey at high concentration in processed foods, infant formula, children snacks especially that manufactured from agriculture materials (e.g. ground peas and maize) to avoid growth of fungi and prevent formation of mycotoxins. According to the results achieved in this paper, a dose of aflatoxin mixture exceeding 1 μg aflatoxins/kg body weigh/day has pronounced harmful effects. This is confirmed by the discussion made recently by the European Union to reduce the maximum limits, of aflatoxins in foods.

## List of abbreviations

**OT group**-group 1, AIN-93 M powder diet fed and administered ochratoxin A (10 ng/kg body weight/day).

**HOT group**-group2, honey AIN-93 M powder diet fed and administered ochratoxin A (10 ng/kg body weight/day).

**AT group**-group 3, AIN-93 M powder diet fed and administered aflatoxins mixture (1 μg/kg body weight/day).

**HAT group**-group 4, honey AIN-93 M powder diet fed and administered aflatoxins mixture (1 μg/kg body weight/day).

**NTC group**-group 5, AIN-93 M powder diet fed.

**HTC group**-group 6, honey AIN-93 M powder diet fed.

## Competing interests

The author(s) declare that they have no competing interests.

## Authors' contributions

Aly Ezz El-Arab conceived and designed the study and performed the colonic microbial enzymes (He also took care of the experimental animal in cooperation with Shenouda Girgis, who examined the chromosomal aberrations). Eman Hegazy carried out the histopathlogical examination and Azzat Abd El-Khalek counted the probiotic bacteria. All the authors reported the data and wrote this manuscript.

## Pre-publication history

The pre-publication history for this paper can be accessed here:


